# Brazilian Academy of Rhinology position paper on topical intranasal therapy

**DOI:** 10.5935/1808-8694.20130067

**Published:** 2015-10-04

**Authors:** João Ferreira de Mello Júnior, Olavo de Godoy Mion, Nilvano Alves de Andrade, Wilma Terezinha Anselmo-Lima, Aldo Eden Cassol Stamm, Washingthon Luiz de Cerqueira Almeida, Pedro Oliveira Cavalcante Filho, Jair de Carvalho e Castro, Francini Grecco de Melo Padua, Fabrizio Ricci Romano, Rodrigo de Paulo Santos, Renato Roitmann, Richard Louis Voegels, Roberto Campos Meirelles, Leonardo Conrado Barbosa Sá, Moacyr Tabasnik Moacyr, Marco Cesar Jorge dos Santos, Roberto Eustáquio Santos Guimarães

**Affiliations:** a Senior Associate Professor - Medical School of the University of São Paulo (FMUSP).; b Professor/Collaborator at the Otorhinolaryngology Program - University of São Paulo.; c PhD in Otorhinolaryngology - Medical School of the University of São Paulo.; d Associate professor - Medical School of Ribeirão Preto - University of São Paulo.; e MSc in Otorhinolaryngology and PhD in Medicine - Federal University of São Paulo.; f Professor/Coordinator of the Otorhinolaryngology Medical Residency Program - Federal University of Rio Grande do Norte.; g PhD in Otorhinolaryngology - Federal University of São Paulo. Head of the Otorhinolaryngology Department - Santa Casa da Misericórdia - Rio de Janeiro.; h PhD in Sciences - FMUSP, Collaborator Physician - Pediatric Otorhinolaryngology Program - Federal University of São Paulo.; i PhD in Sciences - Medical School - University of São Paulo (FMUSP).; j Associate Professor of Otorhinolaryngology - Medical School of the Lutheran University in Brazil. Associate Scientific Staf - Department of Otolaryngology Mount Sinai Hospital Toronto Ontario Canada.; k Senior Associate Professor - State University of Rio de Janeiro.; l MSc in Otorhinolaryngology - Federal University of Rio de Janeiro. Professor/Collaborator - Otorhinolaryngology Program - School of Medical Sciences - State University of Rio de Janeiro.; m Head of the Otorhinolaryngology Service - Policlínica de Botafogo - Rio de Janeiro.; n MD. Otorhinolaryngologist.; o Associate Professor - Medical School of the Federal University of Minas Gerais. Senior Associate Professor - University of São Paulo/Ribeirão Preto.

**Keywords:** medication systems, nasal cavity, nose, therapeutics

## Abstract

This documents aims at educating those who treat sinonasal diseases - both general practitioners and specialists - about topical nasal treatments. By means of scientific evidence reviews, the Brazilian Academy of Rhinology provides its practical and updated guidelines on the most utilized topical nasal medication, except for the drugs that have topical antibiotics in their formulas.

## INTRODUCTION

Inflammatory diseases of the nose and paranasal sinuses are among the most prevalent conditions to affect the general population. Diseases such as allergic and non-allergic rhinitis, acute and chronic rhinosinusitis with and without nasal polyps, cause significant decrease in the quality of life of the affected patients, and adversely affect one's ability to perform activities connected to work, leisure, and socialization. These patients require specific specialized care.

Nasal topical medications are extremely important in the care of patients with inflammatory diseases of the nose and paranasal sinuses and upper airway infections. Although some drug classes have been used for decades, new medications have been made available to patients.

Given the prevalence of these diseases, significant direct and indirect expenditures are associated with treatment, particularly when long term therapies are considered. The costs associated with treatment should not be neglected. Patients, their families, the public health care system and society may experience significant savings as a result of the judicious use of medication.

This paper aims to provide clarification to specialist physicians and general practitioners on the treatment of nose and sinus diseases with topical nasal medication. The Brazilian Academy of Rhinology reviewed the scientific evidence available to offer a practical updated overview on the most commonly used non-antibiotic topical nasal medications.

### Topical intranasal steroids

Intranasal steroids have been effectively used to treat allergic rhinitis, rhinosinusitis, and nasal polyps^1^, in addition to a wide array of non-allergic rhinitis, such as idiopathic, vasomotor, and gestational rhinitis^2^.

Increased knowledge on the pharmacology of glucocorticoid and steroid receptors has enabled the development of molecules designed to potently reach specific localized activity with minimum risk of systemic side effects^3^.

The introduction of topical glucocorticoids and steroids (GCS) has significantly enhanced the treatment of upper and lower airway diseases. Their clinical efficacy may depend partly on the ability to reduce eosinophilic function and infiltration by inhibiting the activation and viability of eosinophils^4^, ^5^, ^6^. They may also act to reduce the release of chemotactic enzymes on the nasal mucosa and polyp epithelial cells^7^. The potency of these drugs is lesser in nasal polyps than in the nasal mucosa, thus suggesting that nose polyps may present induced inflammatory resistance to therapy with steroids^8^.

The biological effect of GCS is mediated by the intracellular activation of glucocorticoid receptors^9^ expressed in most tissues and cells. Two different GCS receptor isoforms have been identified in humans, alpha (GRa) and beta (GRb), both originated in the same gene and divided after the primary transcription of the glucocorticoid receptor^10^. As GRa binds to a hormone, it increases pro-inflammatory genetic transcription and produces most of the anti-inflammatory effect seen in GCS through protein interactions between glucoreceptors and transcription factors such as AP-1 and NF-KB. GRb does not bind to steroids, but may interfere with GRa function. A number of mechanisms are probably involved in resistance to the anti-inflammatory effect provided by GCS, including exaggerated expression of GRb or reduced expression of GRa. Increases in GRb expression have been seen in patients with nasal polyps^11^, ^12^, while downregulation of GRa levels has been reported after treatment with GCS^13^, ^14^ as one of the possible explanations for secondary resistance to GCS^15^. Theoretically, the anti-inflammatory effects of GCS may be seen in allergic and non-allergic rhinosinusitis, for instance in cases of infectious rhinosinusitis; tissue eosinophilia is also found in patients with chronic or persistent rhinosinusitis^16^.

Each glucocorticoid and steroid has unique molecular, pharmacokinetic, and pharmacodynamic properties, which lead to drugs with different modes of action. For example, furoate increases GCS potency and selectivity for the mineralocorticoid receptor^17^. Ciclesonide is an inactive drug converted to a pharmacologically active metabolite, des-isobutyryl-ciclesonide, by upper and lower airway esterases^18^. Ciclesonide and budesonide - the latter not a prodrug - also form fatty acid esters after topical administration on the nasal mucosa (budesonide oleate and budesonide palmitate), thus contributing to their intracellular retention in the nasal mucosa^19^. Pharmacological studies on potency using affinity as the only criterion yielded a classification for GCS. Mometasone furoate, fluticasone furoate, and fluticasone propionate were ranked as the most potent intranasal glucocorticoids and steroids^20^. The side chains in furoate and propionate allow these esters to be highly lipophilic, a quality that may facilitate their absorption by the nasal mucosa and further progression through the cell membrane phospholipase. These compounds do not present significant systemic absorption, with values under 1%, together with ciclesonide^21^.

Studies have shown higher rates of systemic absorption in older GCS, such as beclomethasone dipropionate, triamcinolone acetonide, and budesonide, with bioavailability ranging between 34% and 49%. However, the one-year studies carried out on mometasone, fluticasone propionate, and budesonide to assess the potential systemic side effects in children did not reveal adverse effects on the hypothalamic-pituitary-adrenal axis or upon its growth. In theory, medications with lower levels of bioavailability should be given preference for being closer to the pharmacokinetic and pharmacodynamic traits desired in an ideal course of therapy. However, studies have failed to confirm this assumption^22^, ^23^, ^24^, ^25^, ^26^. These drugs are very useful in managing symptoms and, therefore, can be administered in a chronic regimen (Table 1).

**Table 1:** General characteristics of the formulations of intranasal steroids, age from which they can be used in allergic rhinitis, and corresponding dosages for children and adults.

Name	Formulation	Minimum age	Dose per spray mcg*/nostril	Maximum dose/children mcg/day	Dose/adults mcg/day	Maximum dosage for rhinitis and nasal polyps** mcg/day
Triamcinolone acetonide	Isotonic	4 years	55	110	220	220
Budesonide	Isotonic	6 years	32, 50, 64, 100	100	200	400
Ciclesonide	Hypotonic	6 years	50	100	200	400
Beclomethasone dipropionate	Isotonic	6 years	50	100	200	400
Mometasone furoate	Isotonic	2 years	50	100	200	400
Fluticasone propionate	Isotonic	2 years	50	100	200	400
Fluticasone furoate	Isotonic	4 years	27.5	52.5	105	210

* mcg micrograms. Source: Medication inserts,

** Standard dosages are not available for nose polyps at present; clinical trials usually use the medication's maximum dosage^3^.

Topical intranasal glucocorticoids and steroids are effective against seasonal and perennial allergic rhinitis, non-allergic rhinitis, and episodic rhinitis, as they effectively manage all rhinitis symptoms, including nasal congestion.

Onset of action is usually slower than oral and intranasal antihistamines, occurring within 12 hours in most patients or in three to four hours in some subjects after provocation tests. Full effect takes longer to occur.

When compared to other drug classes, topical GCS are more effective than the combination of oral antihistamines and antileukotriene agents for seasonal or perennial allergic rhinitis. Their efficacy is comparable to that of oral antihistamines for ocular symptoms of allergic rhinitis. Fluticasone furoate has been the most frequently studied drug for ocular symptoms of patients with rhinoconjunctivitis, and was found to be significantly more effective than placebo and just as effective as antihistamines^27^, ^28^.

No systemic side effects have been observed in adults, nor have adverse impact on the growth of children with perennial allergic rhinitis been described, when the drug is administered in the recommended dosages. Local side effects are minimal, but nasal irritation and bleeding may occur. Cases of septal perforation have been reported, although rarely. Drug effect in children and pregnant women is very similar to that observed in adult subjects. However, given the potential unexpected consequences, GCS must be judiciously prescribed and administered to these two populations.

Studies on the use of topical intranasal GCS in children have not described adverse effects or significant levels of systemic absorption. In pregnant women there is always the concern with the embryo and the association with cleft palate, but up to now no teratogenic effects have been reported, despite the few studies done on the matter. Therefore, the risks and benefits provided by these medications must be considered when they are prescribed to pregnant individuals. The only intranasal GCS assigned category B by the Food and Drug Administration for use in pregnant subjects is budesonide^29^. Additionally, the recommendation states that it be used in the lowest dosage possible and for as short as possible. When glaucoma is considered, the literature features reports associating intraocular pressure worsening and use of intranasal GCS. However, other studies have not correlated onset of glaucoma and chronic use of mometasone and ciclesonide. Patients with glaucoma prescribed topical intranasal GCS should be followed by an ophthalmologist until more knowledge on the topic has been gathered.

Clinical trials indicate that there is no difference in efficacy between intranasal GCS medications, but most have compared them to placebo. Few studies have made head-to-head comparisons between GCS medications to assess efficacy.

### Treatment of rhinosinusitis with topical GCS

Many are the indications to use topical intranasal GCS in rhinosinusitis, ranging from acute to chronic disease (Table 2).

**Table 2:** Potential indications for topical nasal glucocorticoids and steroids in cases of rhinosinusitis^3^.

Acute rhinosinusitis

Chronic rhinosinusitis without nasal polyps
Chronic rhinosinusitis with nasal polyps
Postoperative care of chronic rhinosinusitis patients to prevent nasal polyp recurrence
Prophylactic care of patients with recurrent acute rhinosinusitis

Considering rhinosinusitis, many studies have looked into single-drug therapy regimens with GCS and GCS as adjuvant drugs. Authors have studied budesonide^30^, ^31^, ^32^, ^33^, ^34^, ^35^, ^36^, ^37^, ^38^, mometasone^39^, ^40^, fluticasone furoate, fluticasone propionate^41^, ^42^, ^43^, ^44^, ^45^, ^46^, ^47^, and beclomethasone dipropionate^48^, ^49^, ^50^, ^51^, ^52^ and reported improved symptom scores in regimens with antibiotics and significant differences when other criteria were analyzed, such as x-ray images and CT scans, or nasal peak flows and acoustic rhinometry or rhinomanometry. One study reported significant reductions in symptoms of acute rhinosinusitis comparing placebo and antibiotics^53^.

Topical GCS medications have known effects upon nasal polyps and associated symptoms such as obstruction, secretion, and sneezing, although smell is affected to a lesser degree. High level evidence is available on polyp size reduction.

In regards to prevention, low level evidence is available on the prophylactic effects of nasal GCS upon recurrent acute rhinosinusitis.

Scientific knowledge on GCS, their effects on the glucocorticoid receptor and cell transcription processes has grown and improved our understanding of this drug class and its use in therapeutic settings.

Despite the significant amount of information available, the clinical differences between each compound are still not clear. As a drug class, intranasal GCS medications have comparable levels of efficacy in the treatment of upper airway inflammatory diseases.

The commercially available GCS medications are close to reaching the ideal pharmacokinetic and pharmacodynamic properties of this drug class in topical nasal applications, namely:

1. High affinity to the receptor, potency, and specificity to the nasal mucosa
2. Low systemic bioavailability
3. High rate of hepatic clearance and fast systemic elimination
4. Single daily dosage.

However, studies are being carried out to find even better drugs considering the criteria above.

### Topical nasal antihistamines

Intranasal antihistamines may be considered as the first-line treatment for allergic and non-allergic non-infectious rhinitis^54^, ^55^, and are as effective or better than second generation oral antihistamines in the treatment of seasonal allergic rhinitis^56^, ^57^, although they are usually less effective than intranasal GCS in the treatment of allergic rhinitis^58^. However, they have been associated with clinically significant effect upon nasal congestion^59^.

The only intranasal antihistamine currently available in our practice is azelastine, a drug characterized by good efficacy and quick onset of action^60^. Azelastine has been approved for the treatment of seasonal and perennial allergic rhinitis, and showed effect upon nasal congestion, rhinorrhea, sneezing, and nasal itching. It was the first antihistamine associated with significant clinical reduction of nasal congestion^61^, in addition to being the first effectively used in cases of non-allergic rhinitis. However, given their systemic absorption, intranasal antihistamines have been associated with sedation and may inhibit the histamine reaction in skin tests^1^.

Several studies have reported that their efficacy against seasonal rhinitis is greater than or equal to that of second generation oral antihistamines^59^. A systematic review encompassing nine randomized controlled trials comparing intranasal antihistamines and intranasal GCS^62^ concluded that intranasal GCS medications are more effective in managing the nasal symptoms of perennial and seasonal rhinitis. Significant benefits may be yielded when intranasal antihistamines and intranasal GCS are combined^55^, although such combination is not commonly utilized.

Azelastine is formulated as an aqueous solution and administered in the form of a nasal spray with a dosage of 1 mg/ml. It is recommended that each nostril be sprayed twice a day in patients above 12 years of age. Half the dosage is recommended for children aged five and older. Onset of action takes about 15 minutes for significant clinical improvement, a fact that places azelastine as a drug to be used in the early stages of allergy bouts^1^.

Clinical trials on azelastine reported that approximately 19% of the patients complained of bitter taste and 11% reported sleepiness^1^.

Intranasal antihistamines are absorbed by the gastrointestinal tract, and azelastine may thus suppress reactions in the skin test for up to 48 hours^63^.

### Intranasal disodium cromoglycate

Intranasal disodium cromoglycate has been effective for some patients in the prevention and treatment of allergic rhinitis, and has not been associated with side effects. It prevents immediate allergic reactions more than it provides symptom relief after the reaction has begun^64^. It is also used in maintenance allergic rhinitis treatment, with onset of action ranging from four to seven days. Full action may take weeks to occur^65^. In episodic rhinitis, use immediately before exposure to antigens protects subjects for four to eight hours against immediate allergic response^66^.

It acts by inhibiting the degranulation of mast cells, consequently preventing the release of immediate allergic response and allergic inflammation mediators. This drug has a unique mode of action and is known for not being a bronchodilator, antihistamine, or direct anti-inflammatory agent^67^.

The 4% spray solution is indicated for the treatment of seasonal and perennial allergic rhinitis. When used in the treatment of symptoms of seasonal allergic rhinitis, cromoglycate must be started early on in the beginning of the allergy season. Effect is usually noted four to seven days after the start of treatment. However, more severe or perennial cases require two weeks or more until maximum effect sets in. Very symptomatic patients may need combined therapy with antihistamines and/or nasal decongestants during the first days of treatment, given the need of proper contact with the nasal mucosa for the drug to be effective^1^. Treatment must be continued by offering the patient a maintenance dosage that is effective for the remainder of the season or period of exposure to the antigens.

Cromoglycate has been effectively used in the treatment of episodic rhinitis when contact with or exposure to the allergen can be anticipated. In these cases, its onset of action appears to be faster. The protective effect provided by cromoglycate against antigen nasal provocation persists for four to eight hours after administration, thus enabling preventive care when exposure can be predicted, as in the case of veterinarians with allergies.

In controlled trials, cromoglycate was better than placebo. A double-blind randomized placebo-controlled trial with children aged between two and five years of age revealed cromoglycate provided relief from allergic rhinitis symptoms. However, cromoglycate was in general less effective than intranasal GCS, and has not been properly studied in relation to leukotriene antagonists and antihistamines^68^, ^69^, ^70^.

Cromoglycate is a safe medication, Adverse effects are usually mild and local, and include sneezing and sensation of burning. No cases of crust of septal perforation have been described. There is no clinical evidence indicating patients could experience overdosages of cromoglycate.

Given its excellent safety profile and absence of significant interactions with other drugs, cromoglycate may be considered and prescribed to young children and pregnant women^71^, ^72^, thus serving as a valuable alternative when other nasal sprays are contraindicated or not tolerated by the patient.

Proper patient selection is critical when cromoglycate is considered. Reviews have described the limited role cromoglycate has in the treatment and prevention of allergic rhinitis symptoms^71^. There is no evidence that cromoglycate may benefit patients with non-allergic non-infectious rhinitis (NARES) or nasal polyps^72^.

### Nasal decongestants

Nasal congestion is one of the most troubling symptoms for patients with rhinitis^73^. The medications with the best effect on this symptom are nasal decongestants, sympathomimetic drugs that act directly on the capacitance vessels of the turbinates.

They are available in oral (pseudoephedrine and phenylephrine) and intranasal topical (phenylephrine, naphazoline, and oxymetazoline) formulations. Both can produce increased blood pressure, agitation, headaches, anxiety, insomnia, tremor, palpitations, dry mucosa, urinary retention in patients with enlarged prostates, glaucoma worsening, and thyrotoxicosis^2^, ^74^.

Topical vasoconstrictors usually start acting within around 10 minutes. However, when used for more than five or ten days, they may lead to the appearance of drug-induced rhinitis as a consequence of their rebound effect. The ARIA (Allergic Rhinitis and its Impact on Asthma) initiative does not recommend its use on children with allergic rhinitis, and states that adults should limit its use to a maximum of five days^2^.

These drugs must be judiciously used in patients taking monoamine oxidase inhibitors (MAOIs), antihypertensive drugs, digitalis, and L-DOPA.

### Inert cellulose powder

Inert cellulose powder has been used as a thickener in various liquid formulations of nasal application. Natural inert cellulose was recently approved for use in rhinitis patients in Brazil.

Cellulose powder is known to hamper bacterial growth, even though its exact mode of action in allergic rhinitis has not been fully explained. It is believed to form a gelatinous membrane on the epithelium when applied to the nasal mucosa. The gelatinous membrane has greater surface tension than mucus, and acts as a more effective barrier to antigen penetration, which cannot reach the effector cells. Thus, it would not affect symptoms, but the entire allergic process by impacting the development of the allergic inflammatory cascade^75^.

A study carried out on the effects of inert cellulose powder in allergic rhinitis by pollen showed that patients using micronized cellulose powder required significantly less salvage medication to manage nasal symptoms^76^.

Additionally, patients with allergic rhinitis by mites using cellulose powder had fewer nasal symptoms in nasal provocation tests for these antigens^77^.

As it is not a medication, the medical literature on inert cellulose powder is scarce and unclear about its mode of action, action on the mucociliary barrier, and dosage.

### Saline solutions

The use of saline solutions in nasal hygiene has been recommended by specialists, but their effects may go beyond those of an adjuvant therapy^1^, ^2^, ^3^. Saline solution has been indicated for patients with allergic rhinitis, non-allergic rhinitis, acute and chronic rhinosinusitis, and even non-specific conditions such as postnasal drip^1^, ^2^, ^3^.

Our patients have been advised for years to use isotonic (0.9%) sodium chloride solutions. In the 1990's, hypertonic (2% or 3%) sodium chloride solutions were introduced. They were initially used in nose surgery postoperative care to ease the removal of crusts. More recently, the medical literature has reported that these solutions are also useful in controlling nasal symptoms of patients with other clinical conditions such as rhinitis.

Nasal washing with isotonic saline solution is an easy, well tolerated and beneficial procedure with practically no relevant adverse side effects^78^. The most common complaint associated with it is sensation of local irritation, in some cases related to the concentration or conservatives added to the solution.

Benzalkonium chloride is a surfactant belonging to the quaternary ammonium group used to prevent contamination by bacteria and preserve pharmacological activity of topical nasal, ocular, auricular, and cutaneous medication. Its hydrophobic and cationic groups act to increase the permeability of bacterial cell membrane, thus conferring it bactericidal properties^79^, ^80^.

*In vitro* studies have shown that the deleterious effects of benzalkonium chloride on ciliary beat rates decreases with drops in pH. This finding has been observed in pH reductions to 7.4 and even 6.0. As the nasal mucus pH is situated between 5.5 and 6.0, it is reasonable to assume that this fact could interfere with the direct correspondence between in vitro results and physiological conditions of the nasal cavities. There is no concrete evidence to state that benzalkonium chloride hurts the human mucociliary barrier in vivo, but the substance has been accounted for the bitter taste of certain nasal preparations^81^, ^82^.

Nasal washing with hypertonic solution (3%) is safe and carries minimal side effects. A few cases of local irritation, itching, burning, otalgia, and sensation of pressure on the face have been described in the literature^83^.

The exact mode of action of nasal washing with saline solution is still being studied. Some possibilities have been considered, such as mechanical cleaning of the nose, dilution of nasal mucus, induction of rhinorrhea, and effects upon ciliary beat rate and ciliary clearance^83^.

The use of saline solution increases the movements of mucus toward the rhinopharynx by mechanical action. The justification for this hypothesis is based on patient reports of increased efficacy in forced washing cycles, such as in cases in which saline solution is gently injected into the nasal cavity with a syringe. Additionally, when forced washing is performed, crusts are softened and removed more easily. Thick secretion can also become less viscous and be more easily removed^84^.

In nasal diseases, various chemical inflammatory mediators are released and dissolved in mucus, acting directly and indirectly on the mucosa and inducing edema and ciliary beat alterations. Saline solution removes and/or dilutes these mediators, thus reducing local inflammation and, consequently, edema. A reduction on levels of histamine and leukotriene C_4_ has been observed in the nasal washes of patients using hypertonic saline solution^85^. Additionally, the use of hypertonic solution on the nasal mucosa may induce the release of substance P, an important neuropeptide that induces rhinorrhea in a dose-dependent fashion^86^.

Ciliary beating is a relevant defense mechanism of the respiratory tract, and reductions in it have been associated with respiratory diseases such as rhinitis, rhinosinusitis, asthma, and otitis media. In healthy individuals without nasal complaints, the saccharin test (*in vivo*) has shown that hypertonic saline solution (3%) significantly improves ciliary beating, an event not seen with isotonic saline solution^87^.

*In vitro* studies have shown that both isotonic and hypertonic solutions may affect ciliary beat rates. *In vitro* ciliated cells of the nasal mucosa treated with saline solutions in different concentrations (0.06%; 0.12%; 0.9%; 3.0%; 7.0%) only presented altered ciliary beating in hypertonic concentrations^88^. On the other hand, some authors have observed moderate negative impact on ciliary beating in cells treated with isotonic saline solution. When solutions in higher concentrations were used (7%; 14%), ciliary beating ceased after five minutes, a finding reversible only in 7% saline solution^89^.

An *in vitro* study - thus in the absence of the protection offered by nasal mucus - on cultured human nasal epithelial cells compared the effects of hypotonic (0.3%), isotonic, and hypertonic (3.0%) saline solutions on gland secretion and cell morphology. The three solutions did not affect total mucin secretion. However, in the 0.3% and 3.0% concentrations the epithelium was injured, a condition not observed with isotonic saline solution^90^.

Another interesting point concerning nasal washing refers to the pH of the used solution. A double-blind randomized trial found that buffered (pH = 8) and non-buffered hypertonic saline solutions significantly increased mucociliary clearance, without significant differences between the two solutions^91^.

Tonicity of saline solutions was found to affect the absorption of topical medications. The bioavailability of calcitonin applied on the nose in hypotonic or hypertonic saline solutions was four to five times greater than when an isotonic formulation was used^92^. Thus, the cell concentration of ciclesonide is higher when nasal topical ciclesonide is applied with hypotonic saline solution versus isotonic saline solution^93^.

Saline solutions can be applied in three different ways. The simplest involves the use of negative pressure, in which saline solution is poured on the palm of the hand and introduced in the nasal fossae by forced inhalation. Another method employs positive pressure to distribute the fluid in the nasal fossae, such as when syringes or eyedroppers are used. The last option is to use sprays, an easy-to-carry option for patient medication^94^. Each of the possibilities has its pros and cons, but patient compliance must be considered in any of them.

CT scans done after nasal washing with saline solution and ionic contrast have shown greater penetration of the solution into the maxillary and ethmoid sinuses when positive pressure approaches were used than when sprays were employed^94^. Likewise, an analysis of airflow through the different areas of the nasal fossae showed that airflow was very limited in the paranasal sinuses, which are protected by the uncinate process and the middle nasal concha, thus explaining the reduced effectiveness of sprays in reaching these sites^95^.

It is worth mentioning that these approaches refer to the application of saline solution, and not nasal spray medication. For these, it is recommended that patients gently blow their noses before using the topical medication to facilitate penetration. The goal is that the medication makes it to the turbinates, and thus patients have to be instructed to direct the tip of the delivery device to the lateral wall of the nasal cavity^21^. In practical terms, patients are advised to apply the medication on the left nasal fossa with the right hand and vice-versa. As the patient applies pressure onto the device's valve, it is recommended that he/she closes the contralateral nostril with a finger while air is gently inhaled during the application of the medication^96^.

Although nasal hygiene with saline solution is recommended by a number of consensus papers, the literature on the topic is limited. More clinical and laboratory trials are required. Nonetheless, some points must be observed, such as not using cold or hot solutions. Saline solution does not ease symptoms promptly, and patient compliance may become an issue. However, the qualities of saline solution (low cost, few adverse side effects, etc) fully justify its use.
